# The influence of onset of disease on exit from paid employment among workers in The Netherlands: A longitudinal register-based study with 9 years follow-up

**DOI:** 10.5271/sjweh.4064

**Published:** 2022-12-30

**Authors:** Roos W Hijdra, Suzan JW Robroek, Alex Burdorf, Merel Schuring

**Affiliations:** 1 Erasmus University Medical Center Rotterdam, Department of Public Health, Rotterdam, the Netherlands

**Keywords:** cardiovascular disease, common mental disorder, diabetes mellitus, disability benefit, inflammatory disease, psychotic disorder, respiratory disease, unemployment

## Abstract

**Objectives:**

This study investigates the influence of onset of disease on exit from paid employment and whether this differs across diseases and sociodemographic groups.

**Methods:**

Register data from Statistics Netherlands on medication prescription was linked to information on employment status and demographics. Persons who were employed in 2009 and 2010 and who did not use medication for the selected disease in 2009 (N=5 889 036) were followed-up over nine years. Six diseases were identified based on medication prescription in 2010 and 2011: cardiovascular diseases, inflammatory diseases, diabetes mellitus, respiratory diseases, common mental disorders, and psychotic disorders. Four pathways out of paid employment were defined: disability benefits, unemployment, no income, and early retirement. Early exit from paid employment was defined as exiting paid employment before retirement age. Cause-specific Cox proportional hazards regression analyses were performed, with interaction terms for age, sex, and migration background.

**Results:**

Onset of disease increased the likelihood of exit from paid employment, with the strongest associations for psychotic disorders [hazard ratio (HR) 2.88, 95% confidence interval (CI) 2.78–2.98] and common mental disorders (HR 2.00, 95% CI 1.97–2.03). Onset of disease was most strongly associated with disability benefits, followed by unemployment. The influence of common mental and psychotic disorders on disability increased until around middle-age, after which it decreased. The influence of mental health problems on exit from paid employment was stronger for persons with a non-native Dutch background and males.

**Conclusion:**

Onset of diseases, especially mental health disorders, is a risk for exiting paid employment before the retirement age. Effective interventions are needed to enhance an inclusive workforce and prevent involuntary loss of paid employment.

In the past decades, life expectancy has increased and consequently policies have been introduced to prolong working lives (1). Moreover, with the aging workforce the prevalence of health problems among workers has also increased. This is best illustrated in the increase of working life expectancy with disabilities (2). In The Netherlands, 2.3 million persons of working age have a chronic disease (3). It is well-known that health problems influence participation in paid employment (4). However, few studies have addressed the influence of the onset of specific diseases on exit from paid employment.

Several studies have investigated the influence of having a health problem on early exit from paid employment. Early exit from paid employment is defined as exiting paid employment before reaching the retirement age. Both poor self-rated health as well as the presence of specific diseases have been associated with exit from paid employment (4). Longitudinal studies have shown that workers with cardiovascular diseases or diabetes mellitus have an increased risk of early exit from paid employment specifically through disability benefits and early retirement (5, 6). A longitudinal study in 11 European countries reported that exit through disability benefits could be attributed for 18% to the presence of depressive symptoms (7). The extent of interference of a disease with employment depends on the severity and symptoms of the disease (8). For example, for inflammatory diseases – such as rheumatic diseases and inflammatory bowel diseases – research showed that initially most persons will stay employed. However, with longer duration and higher severity of their disease, more persons will exit paid employment (8, 9).

Since the prevalence of specific diseases varies across age, with common mental disorders and inflammatory diseases occurring at a younger age than cardiovascular and respiratory diseases (10, 11), it is of interest to determine the age-dependency of health-based selection into paid employment. The few studies available indicate that older persons with a chronic disease are at higher risk for being out of paid employment (12), and that also, at older age, a chronic disease reduces the likelihood of re-entering paid employment after a period of unemployment (11).

Gender and migration background are demographic characteristics that may influence labor market position of workers with a chronic disease. A Dutch study showed that females were more likely to have a chronic disease (13). Additionally, females with a chronic disease were less likely to be employed compared to males with a chronic disease (10). Other studies that investigated the effect of chronic diseases on exit from paid employment did not find a significant difference between males and females (4, 14). Likewise, a study in The Netherlands showed that persons with a non-native Dutch background were more likely to have a chronic disease compared to native Dutch persons (13), but the contribution of this difference to participation in paid employment remains largely unknown.

Although research clearly shows that having a chronic disease is related to early exit from paid employment, only a few studies (15, 16) have investigated the association between onset of chronic diseases and paid employment. Earlier studies have focused on general health or a specific disease (15–21), an exit pathway (mainly disability benefits), or having a disease instead of onset of diseases (5, 22). By following workers from onset of their diseases onwards, this study ensures that persons who exit paid employment soon after their diagnosis are not missed – which prevents selection bias. In the present longitudinal register-based study with nine years follow-up, we aim to examine: (i) the influence of onset of a variety of diseases on exiting paid employment through different pathways and (ii) whether this differs across groups of age, sex, and migration background.

## Methods

### Study population and design

Longitudinal register data from Statistics Netherlands provided information of all Dutch residents in the period 2009–2019 on employment status, medication use, and sociodemographic factors. The data registries were pseudonymized by Statistics Netherlands with a personal unique number assigned to each Dutch resident. Informed consent was not needed for this study because authorized research institutes are allowed by law to use pseudonymized register-based data for research purposes. The Medical Ethical Committee of Erasmus MC Rotterdam declared that the Medical Research Involving Human Subjects Act does not apply to the current study (MEC-2021-0378).

Persons were included if they were employed for ≥9 months in 2009 as well as 2010, and aged 16–64 years in 2009. In order to ensure onset of disease in 2010 for each disease separately, the total study population was split into six disease related study populations. In these study populations, persons were excluded who used medication in 2009 for cardiovascular diseases (N=606 016), inflammatory diseases (N=1 190 954), diabetes mellitus (N=119 646), respiratory diseases (N=410 319), common mental disorders (N=306 425) or psychotic disorders (N=29 107). In order to avoid temporality of disease, medication use for two consecutive years (2010 and 2011) was required, thereby excluding workers with cardiovascular diseases (N=231 421), inflammatory diseases (N=1066 649), diabetes mellitus (N=20 514), respiratory diseases (N=198 242), common mental disorders (N=169 535), and psychotic disorders (N=20 513). Thus, disease-related study populations consisted of employed persons in 2009 and 2010 who either did or did not use medication in 2010 and 2011 for cardiovascular diseases (N=5 051 599), inflammatory diseases (N=3 631 433), diabetes mellitus (N=5 748 876), respiratory diseases (N=5 280 475), common mental disorders (N=5 413 076) or psychotic disorders (N=5 839 416). Each disease-related study population was split into two groups: the disease group (persons who did develop the specific disease) and the reference group (persons who did not develop a disease). Persons were followed for nine years or until they left paid employment, their medication use changed, or reached retirement age (65 years in The Netherlands).

### Employment status

Statistics Netherlands provided information on the main source of income per month between 2009 and 2019. The transition from employment to different forms of non-employment was investigated for the period 2011–2019. Exit from paid employment was defined as the transition from paid employment into unemployment, disability benefits, early retirement or no income for at least three consecutive months. In The Netherlands, persons receive unemployment benefits or social security benefits after loss of paid employment. A person is eligible for a disability benefit after two years of sickness absence. Early retirement was defined as having a retirement benefit as the main source of income before the age of 65 years. No income concerns persons who did not receive any benefits after exit from paid employment, eg, because they do not receive social security benefits due to the income status of their partner. Early exit from paid employment was defined as exiting paid employment before retirement age. The transition from employment to education was not taken into account in this study because it happened very rarely.

### Medication prescription

Statistics Netherlands provided information on purchased medication that was reimbursed by the health insurance per year between 2009 and 2019. Based on the World Health Organization Anatomical Therapeutic Chemical (ATC) classification codes, the presence of 21 different diseases was identified following the procedure described by Yildiz et al (13) and Huber et al (23). Using these codes, the following six groups of diseases were categorized: cardiovascular diseases, diabetes mellitus, respiratory diseases, common mental disorders (anxiety, depression, sleep disorders), inflammatory diseases, and psychotic disorders (supplementary material, www.sjweh.fi/article/4064, table S1). The presence of a specific disease was dichotomized into having or not having a disease. The onset of a disease was defined as the transition from not having the disease in 2009 into having the disease in 2010 and 2011. Identifying individual diseases within these disease categories or disease severity was not possible with this register data set and the ATC codes. Symptoms are identified as the physical or mental consequences of a specific disease.

### Sociodemographic factors

Register data were obtained on age, sex, educational level, and migration background in 2009. Persons with missing data on education (36.7%) were included in the study population. Educational level was divided in four categories: low, middle, high, and missing. Migration background was based on the country of birth of the parents and was divided into five categories: Dutch, Moroccan, Turkish, Surinamese-Antillean, and other. Age was divided into 5-year intervals.

### Statistical analysis

Firstly, descriptive statistics were used to describe the study population and show the demographic factors and the onset of each disease separately. Secondly, cause-specific Cox proportional hazards regression analyses were performed to investigate the influence of onset of disease on exit from paid employment for each of the pathways. The dependent variable was exit from paid employment into unemployment, disability benefits, no income, or early retirement. The analyses consisted of a separate model for each disease, hence, six models with as independent variables starting with medication for cardiovascular diseases, diabetes mellitus, respiratory diseases, inflammatory diseases, common mental disorders, or psychotic disorders. Within each model those with onset of a particular disease were compared with those without this particular disease. The analyses were adjusted for age, sex, migration background, educational level, and comorbidities. The comorbidities included all other disease categories. Each exit pathway was studied separately as the event of interest, with censoring for the other exit pathways as competing events.

Additionally, we censored in case of missing data for the main source of income for at least three consecutive months, being in education for at ≥3 months, reaching the retirement age (65 years), having a change in medication, and at the end of the follow-up period (December 2019). Interaction analyses were performed by including an interaction term of age×disease, migration background×disease, or sex×disease in the cause-specific Cox proportional hazards model. No interaction analysis was done for education×disease due to the large number of missing values for education. The proportional hazards assumption was examined by assessing log-minus-log plots. The presence of a statistically significant interaction guided our decisions to present stratified analyses or not. The statistical models for interaction are described in the supplementary material. The population attributable fraction (PAF) was calculated using the formula: PAF=Pe(HR-1)/(1+Pe(HR-1)), where Pe is the incidence of a disease in 2010 (24). All analyses were performed using SPSS version 26, (IBM Corp, Armonk, NY, USA).

## Results

**Figure 1:**
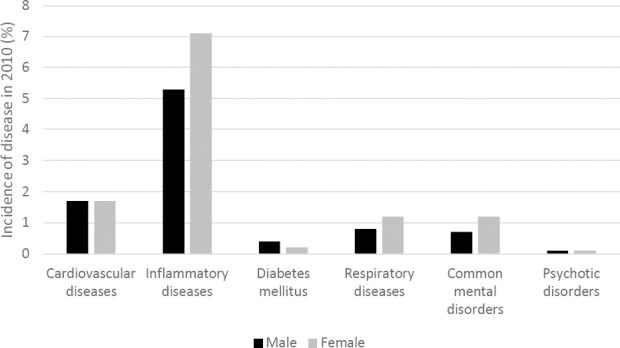
Onset of disease in 2010 among male (black) and female (gray) employees aged 18–65 years in The Netherlands.

The onset of a disease ranged from 6190 (0.1%) for psychotic disorders to 222 139 (6.1%) for inflammatory diseases (figure 1 and supplementary table S2). The mean age of the study population at baseline was 40.7 years [standard deviation (SD) 10.9 years], while the mean age at diagnosis, differed from 40.2 years (SD 10.3 years) among persons with an onset of psychotic disorders to 49.1 years (SD 8.6 years) among persons with an onset of diabetes mellitus. The study population had more males (53.3%) than females, due to higher labor force participation among men at enrolment into the study population of people in paid employment. The gender distribution varied across different diseases, depending on age of onset of disease and proportion of labor force participation in different age groups (supplementary table S2). The incidence of inflammatory diseases, respiratory diseases and common mental disorders was higher among females than males, whereas the incidence of the other diseases was comparable among males and females (cardiovascular diseases and psychotic disorders) or higher among males (diabetes mellitus) (figure 1).

The proportion of exit from paid employment through all pathways was highest among persons with psychotic disorders (49.4%), followed by diabetes mellitus (44.8%) and common mental disorders (34.6%). Exit from paid employment through disability benefits was most common among persons with an incident disease (3.9% for inflammatory diseases to 29.8% for psychotic diseases) compared to the reference groups (range 2.1–3.8%) (supplementary table S3).

Table 1 shows that, after adjustment for age, sex, migration background, educational level, and comorbidities, an onset of disease was associated with exit from paid employment. The likelihood of exit from paid employment was highest among persons with psychotic disorders [hazard ratio (HR) 2.88, 95% confidence interval (CI) 2.78–2.98], followed by common mental disorders (HR 2.00, 95% CI 1.97– 2.03), and cardiovascular diseases (HR 1.28, 95% CI 1.27–1.30). Exit from paid employment was highest in the first year after the onset of a disease and gradually decreased during the following years. After an onset of a psychotic disorder, 85% of the persons were still employed after one year and 55% after four years. After an onset of a cardiovascular disease, approximately 90% of the persons were still employed after one year and approximately 75% after four years (supplementary figure S1). Supplementary figure S2 shows consistency of proportional hazards throughout the nine-year follow up period for all diseases, confirming that the proportional hazard assumption was met.

**Table 1:** The influence of the onset of cardiovascular diseases, inflammatory diseases, diabetes, respiratory diseases, common mental disorders or psychotic disorders on exit from paid employment through different pathways during 9 years follow-up. Analyses were adjusted for age, sex, education, migration background and comorbidity. [HR=hazard ratio; CI=confidence interval.]

	Exit from paid employment (all pathways)	Unemployment	No income	Disability benefits	Early retirement
				
	HR (95% CI)	HR (95% CI)	HR (95% CI)	HR (95% CI)	HR (95% CI)
Cardiovascular diseases (N=5 051 599)	1.28 (1.27–1.30)	1.10 (1.07–1.12)	0.94 (0.91–0.98)	2.68 (2.61–2.75)	1.01 (0.99–1.03)
Inflammatory diseases (N=3 631 433)	1.08 (1.07–1.09)	1.14 (1.13–1.16)	0.75 (0.73–0.77)	2.23 (2.18–2.28)	0.91 (0.89 - 0.94)
Diabetes mellitus (N=5 748 876)	1.12 (1.10–1.15)	1.12 (1.08–1.17)	0.99 (0.92–1.07)	1.50 (1.42–1.59)	0.99 (0.96–1.03)
Respiratory diseases (N=5 280 475)	1.13 (1.11–1.16)	1.11 (1.08–1.14)	0.84 (0.80–0.89)	1.80 (1.73–1.88)	0.97 (0.94–1.01)
Common mental disorders (N=5 413 076)	2.00 (1.97–2.03)	1.65 (1.61–1.69)	1.12 (1.07–1.17)	7.14 (6.96–7.33)	1.06 (1.01–1.11)
Psychotic disorders (N=5 839 416)	2.88 (2.78–2.98)	1.59 (1.49–1.70)	1.21 (1.06–1.38)	8.15 (7.78–8.54)	1.09 (0.92–1.30)

Of the different exit pathways, the onset of a disease was most strongly associated with disability benefits, with HR ranging from 1.50 (95% CI 1.42– 1.59) for diabetes mellitus to 8.15 (95% CI 7.78–8.54) for psychotic disorders. The likelihood to become unemployed was also higher after onset of diseases, with the highest HR for common mental disorders (HR 1.65, 95% CI 1.61–1.69) and psychotic disorders (HR 1.59, 95% CI 1.49–1.70) and lowest for cardiovascular diseases (HR 1.10, 95% CI 1.07–1.12). In addition, persons with an onset of psychotic disorders (HR 1.21, 95% CI 1.06–1.38) or common mental disorders (HR 1.12, 95% CI 1.07–1.17) were more likely to exit paid employment into having no income than persons without these disorders.

In total, 15.9% (95% CI 15.35–16.53) of exit from paid employment through disability benefits could be attributed to onset of any of the six diseases. The proportion of exit from paid employment through disability benefits that could be attributed to a disease was highest for inflammatory diseases (PAF 7.0%, 95% CI 6.7%–7.2%) followed by common mental disorders (PAF 5.5%, 95% CI 5.3%–5.6%) and cardiovascular diseases (PAF 2.8%, 95% CI 2.7%–2.9%). The proportion of exit from paid employment that could be attributed to psychotic disorders was low (PAF 0.8%, 95% CI 0.7%–0.8%), due to the low incidence of psychotic disorders (0.1% in 2010) (supplementary table S4).

Females were more likely to exit paid employment through disability benefits (HR 1.75, 95%CI 1.73–1.77) compared to males. However, among males the onset of the selected six diseases was stronger related to exit from paid employment through both unemployment and disability benefits compared to females. The likelihood of females to become unemployed was slightly lower compared to males (HR 0.98, 95%CI 0.97–0.98). Among males, the onset of cardiovascular diseases was associated with a higher likelihood of exit from paid employment through unemployment (HR 1.20, 95% CI 1.17–1.23), but not among females (HR 0.97, 95% CI 0.94–1.00) (table 2, supplementary table S5).

**Table 2:** The influence of the onset of diseases on exit from paid employment through unemployment and disability benefits among male and female employees. Cox proportional hazard analyses were adjusted for age, migration background, education and comorbidity. [HR=hazard ratio; CI=confidence interval.]

	Male	Female
	
	HR (95% CI) ^a^	HR (95% CI) ^a^
Unemployment		
Cardiovascular diseases	1.20 (1.17–1.23)	0.97 (0.94–1.00)
Inflammatory diseases	1.22 (1.20–1.24)	1.05 (1.03–1.08)
Diabetes mellitus	1.20 (1.15–1.26)	0.99 (0.92–1.05)
Respiratory diseases	1.17 (1.13–1.22)	1.06 (1.02–1.10)
Common mental disorders	1.84 (1.78–1.91)	1.51 (1.46–1.56)
Psychotic disorders	1.71 (1.56–1.87)	1.47 (1.33–1.62)
Disability benefits		
Cardiovascular diseases	3.96 (3.81–4.10)	1.95 (1.88–2.03)
Inflammatory diseases	2.66 (2.57–2.75)	1.94 (1.88–2.00)
Diabetes mellitus	1.81 (1.69–1.94)	1.17 (1.07–1.28)
Respiratory diseases	2.34 (2.20–2.49)	1.52 (1.44–1.61)
Common mental disorders	9.86 (9.49–10.24)	5.95 (5.76–6.15)
Psychotic disorders	10.28 (9.64–10.97)	6.70 (6.27–7.16)

a Statistically significant difference between males and females, + Reference group is males.

The associations between the onset of common mental and psychotic disorders with disability benefits also increased until 35–39 years, after which the magnitude of the associations decreased again (figure 2a). The association between onset of all diseases and unemployment slowly decreased between ages 25–29 and 50–55 years, after which the associations between the onset of psychotic disorders and cardiovascular diseases with unemployment slightly increased with age (figure 2b). Among each age group, exit from paid employment was highest in the first year after an onset of disease and gradually decreased during the following years. (supplementary figure S3)

**Figure 2a:**
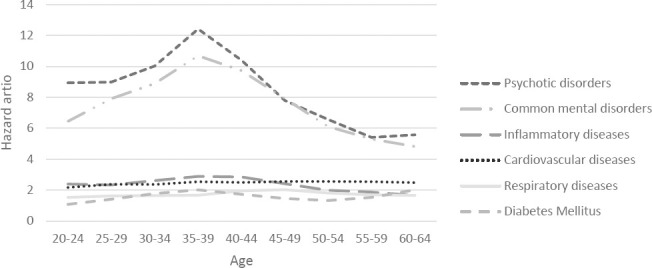
The influence of the onset of a disease on exit from paid employment through disability among 5-year age groups (20-60 years)

**Figure 2b:**
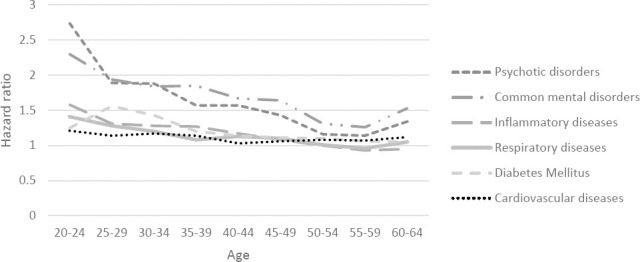
The influence of the onset of a disease on exit from paid employment through unemployment among 5-year age groups (20-60 years).

Moroccan (HR 1.39, 95% CI 1.37–1.41), Turkish (HR 1.45, 95% CI 1.43–1.48), Surinamese/Antillean (HR 1.46, 95% CI 1.44–1.48), and persons with another non-Dutch background (HR 1.37, 95% CI 1.36–1.38) were more likely to become unemployed compared to native Dutch persons. However, table 3 shows that the onset of cardiovascular diseases increased the likelihood of exit from paid employment through unemployment (HR 1.15, 95% CI 1.13–1.18) among native Dutch persons but not among persons with migration backgrounds (HR 0.74–0.99) (table 3, supplementary table S6).

**Table 3:** The influence of the onset of diseases on exit from paid employment through unemployment and disability benefits among employees with different migration backgrounds. Cox proportional hazards analyses, adjusted for age, sex, education and comorbidity. [HR=hazard ratio; CI=confidence interval.]

	Native Dutch	Moroccan	Turkish	Surinamese-Antillean	Other
				
	HR (95% CI)	HR (95% CI)	HR (95% CI)	HR (95% CI)	HR (95% CI)
Unemployment					
Cardiovascular diseases	1.15 (1.13–1.18) ^a^	0.75 (0.60–0.94) ^a^	0.83 (0.71–0.98) ^a^	0.74 (0.67–0.81) ^a^	0.99 (0.94–1.04) ^a^
Inflammatory diseases	1.18 (1.16–1.20)	1.02 (0.93–1.12) ^a^	0.99 (0.92–1.07) ^a^	0.97 (0.91–1.04) ^a^	1.08 (1.04–1.12) ^a^
Diabetes mellitus	1.19 (1.14–1.24)	1.03 (0.82–1.28)	1.17 (0.96–1.42)	0.93 (0.82–1.06) ^a^	1.00 (0.90–1.10) ^a^
Respiratory diseases	1.12 (1.09–1.16)	1.23 (1.00–1.52)	1.29 (1.09–1.52)	1.05 (0.92–1.20)	1.02 (0.94–1.10) ^a^
Common mental disorders	1.63 (1.58–1.67)	2.11 (1.86–2.39) ^a^	1.83 (1.63–2.06) ^a^	1.46 (1.28–1.66)	1.66 (1.56–1.77)
Psychotic disorders	1.67 (1.54–1.80)	1.19 (0.81–1.74)	1.55 (1.15–2.10)	1.35 (0.99–1.85)	1.42 (1.17–1.73)
Disability benefits					
Cardiovascular diseases	2.80 (2.72–2.88) ^a^	1.79 (1.46–2.18) ^a^	2.10 (1.80–2.44) ^a^	1.88 (1.64–2.16) ^a^	2.61 (2.41–2.82)
Inflammatory diseases	2.21 (2.15–2.27)	2.34– (2.09–2.62)	2.11 (1.91–2.33)	2.10 (1.88–2.34)	2.39 (2.45–2.55) ^a^
Diabetes mellitus	1.60 (1.50–1.71)	1.42 (1.10–1.83)	1.24 (0.98–1.57) ^a^	1.14 (0.91–1.43) ^a^	1.36 (1.15–1.59)
Respiratory diseases	1.86 (1.77–1.95)	1.29 (0.98–1.69)	1.58 (1.29–1.94)	1.76 (1.44–2.14)	1.69 (1.50–1.90)
Common mental disorders	6.63 (6.43–6.83)	11.00 (9.93–12.19) ^a^	10.11 (9.24–11.07) ^a^	8.21 (7.33–9.20) ^a^	7.75 (7.23–8.31) ^a^
Psychotic disorders	7.80 (7.36–8.26)	8.89 (7.52–10.50)	8.66 (7.48–10.04)	7.68 (6.20–9.51)	9.61 (8.46–10.92) ^a^

a Statistically significant difference compared to native Dutch employees + Reference group is native Dutch

Regarding the exit through disability, Moroccan (HR 2.76, 95% CI 2.69–2.84), Turkish (HR 2.49, 95% CI 2.43–2.56), Surinamese/Antillean (HR 1.27, 95% CI 1.23–1.31), and persons with other non-Dutch background (HR 1.26, 95% CI 1.24–1.28) were more likely to become disabled compared to native Dutch persons. In addition, the onset of common mental disorders increased the likelihood of exit from paid employment through disability benefits more strongly among persons with a non-native Dutch background (HR 7.75–11.00) than native Dutch persons (HR 6.63, 95% CI 6.43–6.83) (table 3, supplementary table S6).

## Discussion

This register-based study showed that an onset of disease increased the likelihood of exit from paid employment, with the strongest associations for psychotic disorders (HR 2.9), followed by common mental disorders (HR 2.0), and cardiovascular diseases (HR 1.3). Workers lost paid employment largely through entering disability benefits, and to a smaller extent, unemployment. The onset of common mental and psychotic disorders also increased the likelihood of exit from paid employment into no income and early retirement. The impact on population level was highest for inflammatory diseases (PAF 7.0%) and common mental disorders (PAF 5.5%), and lowest for psychotic disorders (PAF 0.8%) and respiratory diseases (PAF 0.8%). The influence of the onset of a disease on exit from paid employment through disability benefits increased until middle age for both common mental and psychotic disorders, after which it decreased again. Among males, the onset of a disease had a stronger association with unemployment and disability benefits than among females. Among workers with a non-native Dutch migration background, the onset of common mental disorders had a stronger influence on disability benefits compared to workers with a native Dutch background.

This study showed consistently that the onset of a disease had adverse consequences on maintaining paid employment. This finding corroborates results of earlier studies on the relation between having a disease and exit from paid employment (4). The finding that workers with an onset of common mental and psychotic disorders had the highest likelihood of exit from paid employment is in line with a study on Norwegian register data showing that – after workers with musculoskeletal disorders – workers with mental disorders had the highest likelihood of exiting paid employment through disability benefits (25). Furthermore, this study showed that persons with common mental disorders were also more likely to exit with no income. Some persons with common mental or psychotic disorders may experience more difficulties applying for unemployment or disability benefits due to problems with cognitive or other psychological limitations. This may cause them to end up with no income (26).

Estimations of the impact on population level of the onset of a disease on exit from paid employment was done by calculating the PAF, combining the incidence of the disease with the magnitude of the association between onset of disease and quitting paid employment. Although psychotic disorders had the highest HR, it had one of the lowest PAF due to the low incidence of psychotic disorders. Contrarily, inflammatory diseases had the highest PAF because the incidence of inflammatory diseases is high compared to the other diseases. This could be due to the fact that inflammatory diseases included a broad range of diseases, such as eczema, inflammatory bowel disease, and asthma, whereby persons often experience prolonged periods of remission interspersed with re-starting their medication (27, 28).

The differences between diseases in the magnitude of the associations with exit from paid employment may be explained by differences in the impact of a specific disease on a person’s life. Our study showed that an onset of common mental and psychotic disorders resulted in the highest likelihood of exiting paid employment. Symptoms of common mental disorders vary from depressed mood and fatigue to anger, anxiety, and suicidal thoughts (29). A study among workers in The Netherlands showed that when comparing mental and physical disorders, persons with mental disorders had less qualitative functioning and a higher number of total workdays lost. This was most often due to concentration difficulties (30). Furthermore, symptoms of mental disorders are often related to behavioral, cognitive, emotional and inter-personal functioning (25). This could impose greater difficulties in employment because such symptoms can be more difficult to change and for coworkers to understand and deal with (25). In addition, persons with mental disorders also have to deal with stigma. Stigma at the workplace can cause adverse work outcomes. This can be due to stigmatization from the employer or co-workers but also self-stigmatization of the worker. Stigmatization can lead to exclusion of the worker or making negative assumptions about the capabilities of the worker (31).

This study showed that the magnitude of the association between the onset of mental disorders and exit from paid employment differs across the life course with a peak around middle age. Knudsen et al (25) showed that compared to persons with other diseases, persons with mental disorders received a disability benefit 6.9 years earlier, resulting in a higher number of working years lost (25). Common mental and psychotic disorders often occur at a younger age. Additionally, certain mental disorders (developmental disorders, intellectual disability, etc.) decrease working capacity significantly. This could imply that these people may never return to paid employment (25). When persons exit paid employment early in working life, it causes more working years lost, having a major impact on both the individual (32–35) and societal level (36).

The influence of disease on labor force participation differed across workers with different migration backgrounds. The effects of common mental or psychotic disorders on exit from paid employment were more profound among persons with a non-native Dutch background compared to native Dutch persons. Harber-Aschan et al (37) also found that in Sweden persons with a migrant background and common mental disorders were more likely to exit paid employment through disability benefits and unemployment compared to native Swedish persons with common mental disorders (37). This may be due to cultural differences. Two Dutch studies suggested that cultural differences may play a role, with migrants being less willing to talk about mental health issues. This may result in underutilization of mental health care with persons only seeking help when problems are very severe, increasing the likelihood of exit from paid employment. Additional barriers in seeking and receiving the accurate healthcare are language barriers and lack of knowledge about the mental healthcare system. Lack of timely and appropriate treatment in mental healthcare can cause a deterioration in mental health and eventually lead to exit from paid employment (38, 39).

This study also showed differences between males and females. The onset of a disease was more strongly associated with exit from paid employment among males compared to females. Yet, overall, females have a higher incidence of disease and were more likely to exit paid employment. This is in line with research from De Boer et al (40) where they compared employees with and without a disease in The Netherlands (40). A study performed in Denmark found that the risk of receiving disability benefits increased with age, with a steeper increase for males than females (41). The difference between sex in the incidence of disease and the risk of exiting paid employment could be due to the higher care-seeking behavior among females. Males may seek healthcare later and with more severe symptoms (42), which can cause a specific disease to have a greater impact on the working life of males (43).

This register based study has strengths and limitations. A strength is that information of a large group is available and monthly tax information provides detailed information on employment status. The large study population captures the population of The Netherlands, without problems of selective participation. Another strength of this study is the 9-year follow-up period. This enabled us to show the long-term effects of the onset of a disease on exit from paid employment. Furthermore, the current study gives a more objective insight into the association between diseases and employment than self-reported data. Because this study focuses on the onset of diseases, which occurred in the year before the start of the follow-up period, reversed causality was ruled out.

However, several limitations need to be mentioned. Firstly, with the register information on medication, only persons were included who had a particular drug prescribed, purchased this drug, and had it reimbursed by the health insurer. If one of these steps was missing, this person was not identified as having a disease. A linked issue is that medication use was converted into broad disease categories, with some inevitable misclassification. Furthermore, register information on prescribed medication is by default limited to persons who use medication, thus, other forms of treatments (eg, behavioral therapy) cannot be identified. This will cause an underestimation of the incidence of diseases. Diseases that often are treated with over-the-counter drugs are also not identifiable. Because this register dataset does not provide information on prescribed quantities of medication, disease severity or complications associated with the disease cannot be identified. Each disease category includes a variety of diseases with different symptoms. Additionally, information about medication was available on a yearly basis. We also cannot identify whether the person actually took the medication. Lastly, because of the large number of missing information for educational level, differences across educational groups were not studied.

### Concluding remarks

This study showed that the onset of a disease is associated with exit from paid employment, in particular due to disability benefits and – to a smaller extent – unemployment. Future policies should therefore focus not only on the prevention of health problems, but also on inclusiveness of the labor market in order to prevent involuntarily exit from paid employment for persons with a disease.

## Supplementary material

Supplementary material
