# Supplementary material for: The influence of onset of disease on exit from paid employment among workers in The Netherlands: A longitudinal register-based study with 9 years follow-up
Source: Scand J Work Environ Health. 2022 Dec 30;49(1):33–42. doi: 10.5271/sjweh.4064 (PMC10549911; doi:10.5271/sjweh.4064)
Supplement: Supplementary material [file SJWEH-49-33-S001.pdf]

The influence of onset of disease on exit from paid employment among workers in The Netherlands: A longitudinal register-based study with 9 years follow-up[1]

by Roos W Hijdra, MSc, Suzan JW Robroek, PhD, Alex Burdorf, PhD, Merel Schuring, PhD[2]

1. Supplementary material
2. Correspondence to: M Schuring, Erasmus University Medical Center Rotterdam, Department of Public Health, PO Box 2040, 3000 CA Rotterdam, the Netherlands. [E-mail: M.schuring@erasmusmc.nl]

Table S1 Description of ATC codes used to identify diseases among the study population

Chronic disease*	ATC-code	Medication class
**Cardiovascular diseases**	B01A<br>C01<br>C03A<br>C08<br>C07<br>C09A, C09B	Antithrombotic agents<br>Cardiac agents<br>Low-ceiling drugs<br>Calcium channel blockers<br>Beta blocking agents<br>ACE inhibitors
**Common mental disorders (anxiety, depression, sleep disorders)**	N05B<br>N05C<br>N06A	Anxiolytics<br>Hypnotics and sedatives<br>Antidepressants
**Inflammatory conditions**	M01A	Anti-inflammatory and antirheumatic products, non-steroids
**Respiratory illness**	R03A, R03C<br>R03B, R03D	Adrenergics (inhalants)<br>Other drugs for obstructive airway diseases
**Psychotic disorders (psychoses)**	N05A	Antipsychotics
**Diabetes mellitus**	A10A<br>A10B	Insulins and analogues<br>Blood glucose lowering drugs

*These chronic diseases were identified using the ATC-codes of prescribed medication following the study of Huber on identification of chronic diseases by ATC codes (1) and the study of Van Ooijen (2).

1. Huber CA, Szucs TD, Rapold R, Reich O. Identifying patients with chronic conditions using pharmacy data in Switzerland: an updated mapping approach to the classification of medications. BMC Public Health. 2013;13:.
2. van Ooijen R. Life cycle behavior under uncertainty: Essays on savings, mortgages and health. Groningen: University of Groningen, SOM research school; 2016.

Table S2 Individual characteristics of employed persons (N=5 889 036) with cardiovascular diseases, inflammatory diseases, diabetes, respiratory diseases, common mental disorders or psychotic disorders

		Study population N=5 899 036	Cardiovascular diseases N=86 425*	Inflammatory diseases N=222 139*	Diabetes Mellitus N=16 340*	Respiratory diseases N=51 260*	Common mental disorders N=50 851*	Psychotic disorders N=6 190*
Mean age (SD)	Male	41.1 (11.1)	49.0 (8.6)	42.7 (10.7)	49.7 (8.3)	43.7 (10.9)	42.9 (10.2)	40.9 (10.3)
	Female	40.3 (10.7)	54.6 (9.6)	41.2 (10.6)	48.0 (9.0)	41.7 (10.7)	40.6 (10.3)	39.4 (10.2)
		N (%)	N (%)	N (%)	N (%)	N (%)	N (%)	N (%)
Sex	Male	3 141 310 (53.3)	45 738 (52.9)	106 827 (48.1)	10 712 (65.6)	22 996 (44.9)	21 256 (41.8)	3 184 (51.4)
	Female	2 747 694 (46.7)	40 686 (47.1)	115 311 (51.9)	5 628 (34.4)	28 264 (55.1)	29 595 (58.2)	3 006 (48.6)
Migration background	Dutch	4 900 858 (83.2)	72 773 (84.2)	180 107 (81.1)	12 247 (75.0)	41 922 (81.8)	40 183 (79.0)	4 606 (74.4)
	Moroccan	71 214 (1.2)	655 (0.8)	4 090 (1.8)	354 (2.2)	686 (1.3)	1 402 (2.8)	257 (4.2)
	Turkish	92 243 (1.6)	1 068 (1.2)	5 792 (2.6)	431 (2.6)	971 (1.9)	1 858 (3.7)	339 (5.5)
	Surinamese Antillean	164 276 (2.8)	2 988 (3.5)	7 848 (3.5)	1 193 (7.3)	1 836 (3.6)	1 638 (3.2)	252 (4.1)
	Other	660 445 (11.2)	8 941 (10.3)	24 302 (10.9)	2 115 (12.9)	5 845 (11.4)	5 770 (11.3)	736 (11.9)

*Persons with a diagnosis of the selected disease in 2010

Table S3 Exit from paid employment through different pathways among employed persons with or without the onset of cardiovascular diseases, inflammatory diseases, diabetes, respiratory diseases, common mental disorders and psychotic disorders

	Exit from paid employment during follow-up (N, %)	Unemployment (N, %)	No income (N, %)	Disability benefits (N, %)	Early retirement (N, %)
Cardiovascular diseases					
Onset of disease (N=86 425)	30 039 (34.8)	11 011 (12.7)	3 181 (3.7)	5 789 (6.7)	10 058 (11.6)
Reference group (N=4 965 174)	1 392 169 (28.0)	749 138 (15.1)	281 929 (5.7)	134 144 (2.7)	226 958 (4.6)
Inflammatory diseases					
Onset of disease (N=222 139)	43 702 (19.7)	21 384 (9.6)	6 055 (2.7)	8 700 (3.9)	7 563 (3.4)
Reference group (N=3 409 294)	891 853 (26.2)	439 435 (12.9)	184 963 (5.4)	71 797 (2.1)	195 658 (5.7)
Diabetes Mellitus					
Onset of disease (N=16 340)	7 323 (44.8)	2 817 (17.2)	746 (4.6)	1 276 (7.8)	2 484 (15.2)
Reference group (N=5 732 536)	1 878 482 (32.8)	936 071 (16.3)	340 450 (5.9)	218 254 (3.8)	383 707 (6 7)
Respiratory diseases					
Onset of disease (N=51 260)	11 301 (22.0)	5 013 (9.8)	1 419 (2.8)	2 291 (4.5)	2 578 (5.0)
Reference group (N=5 229 215)	1 641 797 (31.4)	816 661 (15.6)	303 444 (5.8)	176 121 (3.4)	345 571 (6.6)
Common mental disorders					
Onset of disease (N=50 851)	17 595 (34.6)	7 056 (13.9)	1 789 (3.5)	7 067 (13.9)	1 683 (3.3)
Reference group (N=5 362 225)	1 596 407 (29.8)	804 048 (15.0)	303 050 (5.7)	127 943 (2.4)	361 366 (6.7)
Psychotic disorders					
Onset of disease (N=6 190)	3 056 (49.4)	861 (13.9)	217 (3.5)	1 846 (29.8)	132 (2.1)
Reference group (N=5 833 226)	1 916 248 (32.9)	950 067 (16.3)	344 467 (5.9)	207 994 (3.6)	413 720 (7.1)

Table S4 The proportion of exit from paid employment through unemployment and disability that can be attributed to cardiovascular diseases, inflammatory diseases, diabetes, respiratory diseases, common mental disorders or psychotic disorders

Unemployment							
	Incidence	HR			PAF		
	%		L 95% CI	H 95% CI		L95%CI	H95%CI
Cardiovascular diseases	0.017	1.1	1.07	1.12	0.0017	0.0012	0.0020
Inflammatory diseases	0.061	1.14	1.13	1.16	0.0085	0.0079	0.0097
Diabetes Mellitus	0.0028	1.12	1.08	1.17	0.0003	0.0002	0.0005
Respiratory diseases	0.0097	1.11	1.08	1.14	0.0011	0.0008	0.0014
Common mental disorders	0.0094	1.65	1.61	1.69	0.0061	0.0057	0.0064
Psychotic disorders	0.0011	1.59	1.49	1.7	0.0006	0.0005	0.0008
Disability							
	Incidence	HR			PAF		
	%		L 95% CI	H 95% CI		L95%CI	H95%CI
Cardiovascular diseases	0.017	2.68	2.61	2.75	0.0278	0.0266	0.0289
Inflammatory diseases	0.061	2.23	2.18	2.28	0.0698	0.0671	0.0724
Diabetes Mellitus	0.0028	1.5	1.42	1.59	0.0014	0.0012	0.0016
Respiratory diseases	0.0097	1.8	1.73	1.88	0.0077	0.0070	0.0085
Common mental disorders	0.0094	7.14	6.96	7.33	0.0546	0.0531	0.0562
Psychotic disorders	0.0011	8.15	7.78	8.54	0.0078	0.0074	0.0082

Table S5 The influence of the onset of diseases on exit from paid employment through unemployment and disability benefits among male and female employees

Unemployment		
	Male (HR, 95% CI)	Female (HR, 95% CI)
Sex	1	0.98 (0.97-0.98)
Cardiovascular diseases	1.20 (1.17-1.23)	0.97 (0.94-1.00)*
Inflammatory diseases	1.22 (1.20-1.24)	1.05 (1.03-1.08)*
Diabetes Mellitus	1.20 (1.15-1.26)	0.99 (0.92-1.05)*
Respiratory diseases	1.17 (1.13-1.22)	1.06 (1.02-1.10)*
Common mental disorders	1.84 (1.78-1.91)	1.51 (1.46-1.56)*
Psychotic disorders	1.71 (1.56-1.87)	1.47 (1.33-1.62)*
Disability benefits		
Sex	1	1.75 (1.73-1.77)
Cardiovascular diseases	3.96 (3.81-4.10)	1.95 (1.88-2.03)*
Inflammatory diseases	2.66 (2.57-2.75)	1.94 (1.88-2.00)*
Diabetes Mellitus	1.81 (1.69-1.94)	1.17 (1.07-1.28)*
Respiratory diseases	2.34 (2.20-2.49)	1.52 (1.44-1.61)*
Common mental disorders	9.86 (9.49-10.24)	5.95 (5.76-6.15)*
Psychotic disorders	10.28 (9.64-10.97)	6.70 (6.27-7.16)*

Cox proportional hazard analyses were adjusted for age, migration background, education and comorbidity. * Statistically significant difference between males and females

Model specification for calculation and interpretation of interaction terms

An interaction term of sex*disease was used to investigate differences in associations between the sexes. The following Cox proportional Hazards model was used y_{it}= b1(females) + b2(disease) + b3(females*disease) + confounders. With b1, the influence of being female on exit from paid employment at time t is estimated. With b2, the influence of the disease among males on exit from paid employment is estimated. B3 represents the difference in the influence of the disease among females compared to males. With b2+b3, the influence of the disease among females on exit from paid employment is estimated.

Table S6 The influence of the onset of diseases on exit from paid employment through unemployment and disability benefits among employees with different migration backgrounds

	Native Dutch (HR, 95% CI)	Moroccan (HR, 95% CI)	Turkish (HR, 95% CI)	Surinamese-Antillean (HR, 95% CI)	Other (HR, 95% CI)
Unemployment					
Migration background	1	1.39 (1.37-1.41)	1.45 (1.43-1.48)	1.46 (1.44-1.48)	1.37 (1.36-1.38)
Cardiovascular diseases	1.15 (1.13-1.18)*	0.75 (0.60-0.94)*	0.83 (0.71-0.98)*	0.74 (0.67-0.81)*	0.99 (0.94-1.04)*
Inflammatory diseases	1.18 (1.16-1.20)	1.02 (0.93-1.12)*	0.99 (0.92-1.07)*	0.97 (0.91-1.04)*	1.08 (1.04-1.12)*
Diabetes Mellitus	1.19 (1.14-1.24)	1.03 (0.82-1.28)	1.17 (0.96-1.42)	0.93 (0.82-1.06)*	1.00 (0.90-1.10)*
Respiratory diseases	1.12 (1.09-1.16)	1.23 (1.00-1.52)	1.29 (1.09-1.52)	1.05 (0.92-1.20)	1.02 (0.94-1.10)*
Common mental disorders	1.63 (1.58-1.67)	2.11 (1.86-2.39)*	1.83 (1.63-2.06)*	1.46 (1.28-1.66)	1.66 (1.56-1.77)
Psychotic disorders	1.67 (1.54-1.80)	1.19 (0.81-1.74)	1.55 (1.15-2.10)	1.35 (0.99-1.85)	1.42 (1.17-1.73)
Disability benefits					
Migration background	1	2.76 (2.69-2.84)	2.49 (2.43-2.56)	1.27 (1.23-1.31)	1.26 (1.24-1.28)
Cardiovascular diseases	2.80 (2.72-2.88)*	1.79 (1.46-2.18)*	2.10 (1.80-2.44)*	1.88 (1.64-2.16)*	2.61 (2.41-2.82)
Inflammatory diseases	2.21 (2.15-2.27)	2.34- (2.09-2.62)	2.11 (1.91-2.33)	2.10 (1.88-2.34)	2.39 (2.45-2.55)*
Diabetes Mellitus	1.60 (1.50-1.71)	1.42 (1.10-1.83)	1.24 (0.98-1.57)*	1.14 (0.91-1.43)*	1.36 (1.15-1.59)
Respiratory diseases	1.86 (1.77-1.95)	1.29 (0.98-1.69)	1.58 (1.29-1.94)	1.76 (1.44-2.14)	1.69 (1.50-1.90)
Common mental disorders	6.63 (6.43-6.83)	11.00 (9.93-12.19)*	10.11 (9.24-11.07)*	8.21 (7.33-9.20)*	7.75 (7.23-8.31)*
Psychotic disorders	7.80 (7.36-8.26)	8.89 (7.52-10.50)	8.66 (7.48-10.04)	7.68 (6.20-9.51)	9.61 (8.46-10.92)*

Cox proportional hazards analyses, adjusted for age, sex, education and comorbidity.

* Statistically significant difference compared to native Dutch employees

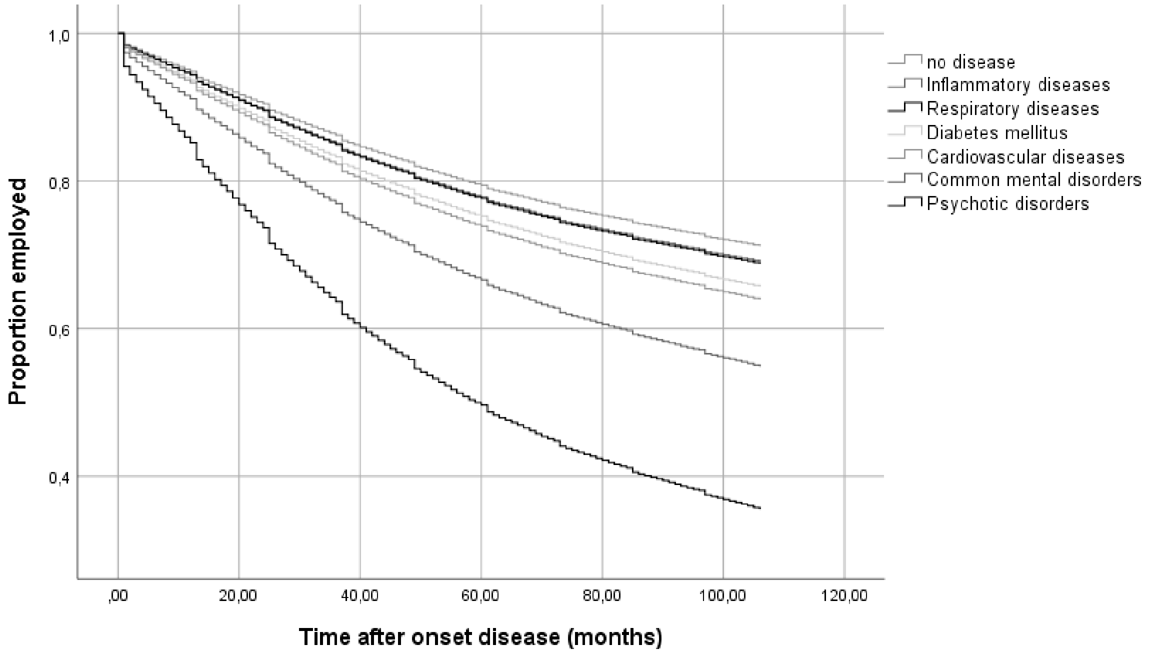

Figure S1 Proportion of persons in employment after the onset of a disease (survival curve based on the adjusted cox proportional hazards model for exit from paid employment through all pathways)

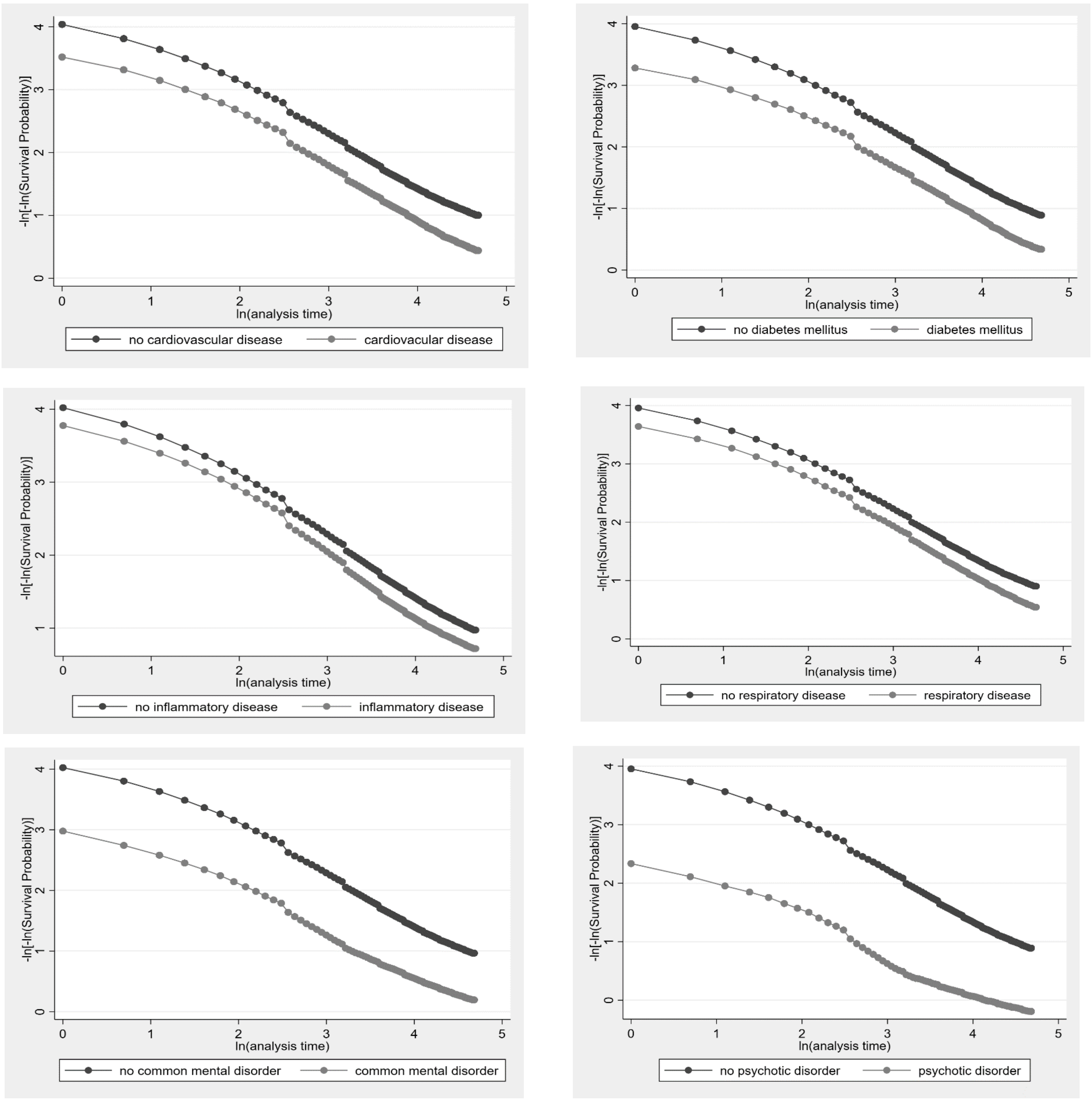

Figure S2 Log-Log plot for Goodness of Fit with Proportional Hazards Assumption

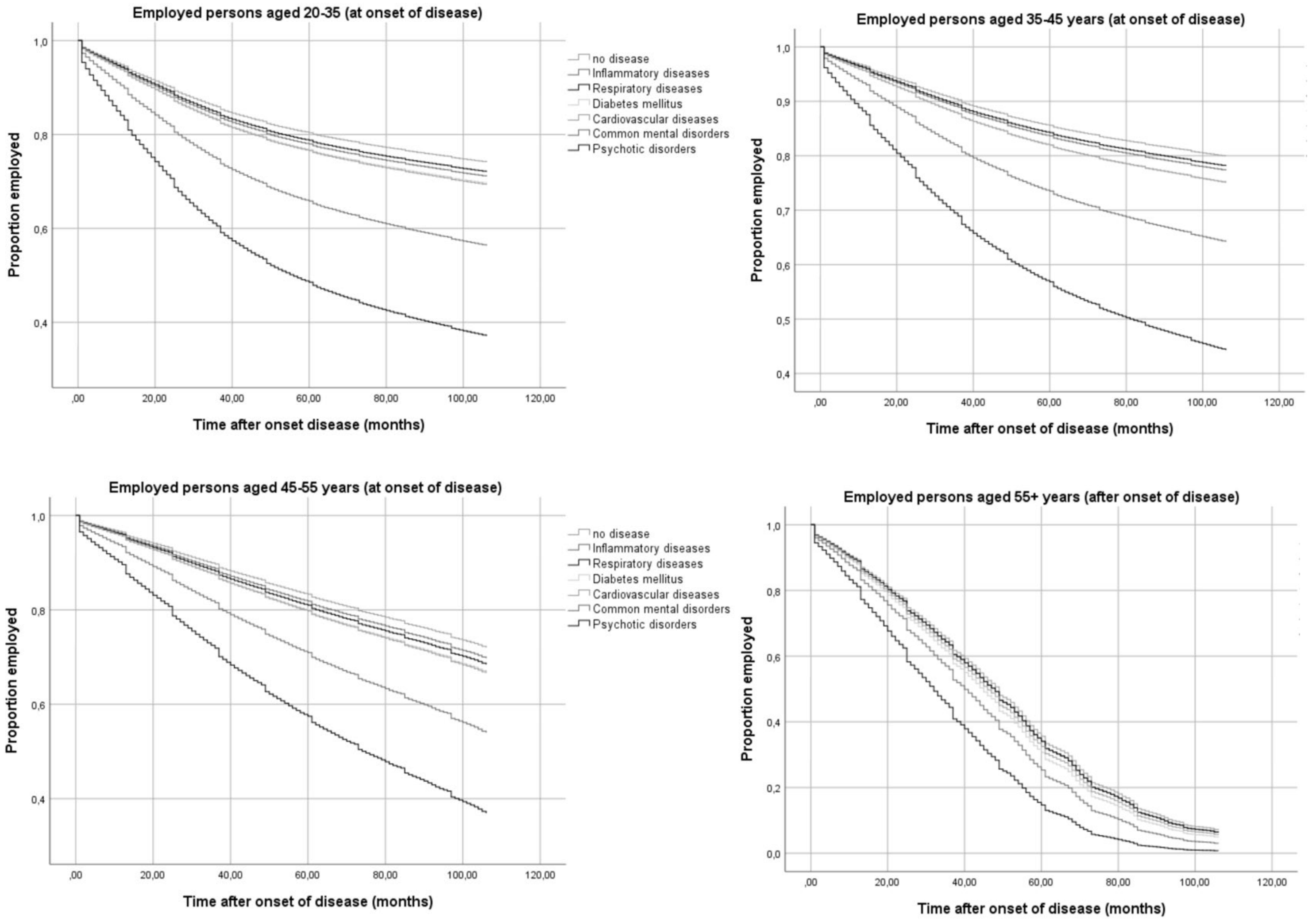

Figure S3 Proportion of persons in employment after the onset of disease at different ages (survival curves based on the adjusted cox proportional hazards model for exit from paid employment through all pathways)

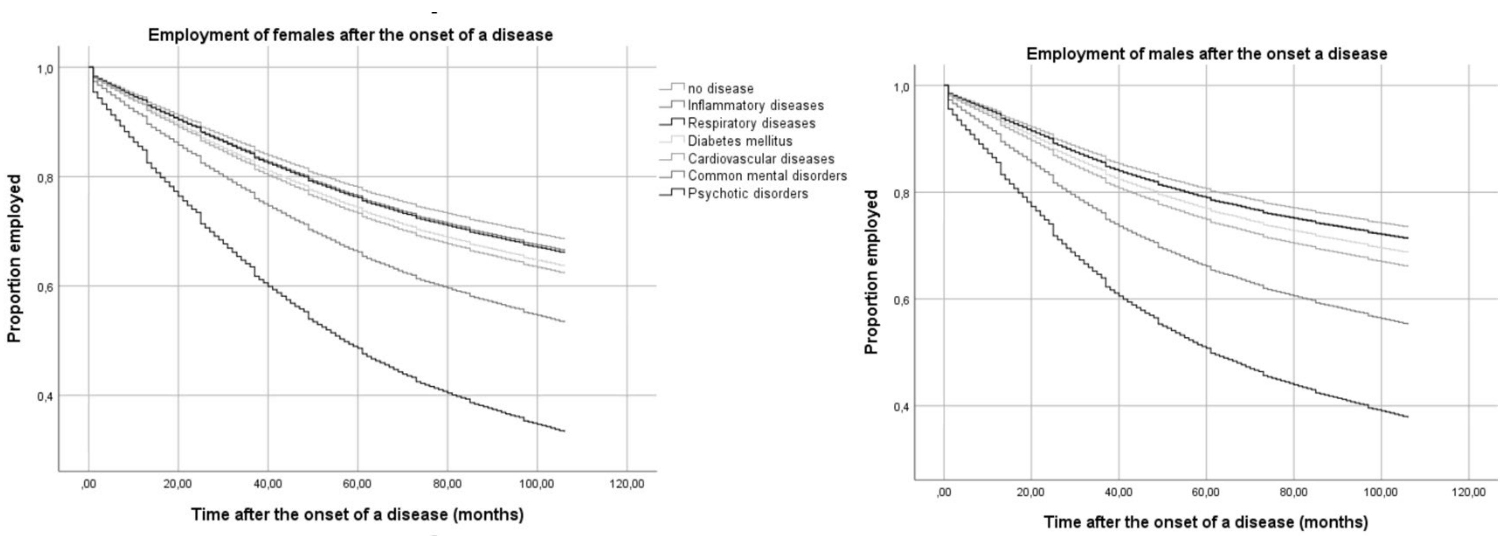

Figure S4 Employment among females and males after the onset of a disease (survival curves based on the adjusted cox proportional hazards model for exit from paid employment through all pathways)
